# The Impact of Patient, Tumor, and Socioeconomic Characteristics on Survival in Upper Urinary Tract Urothelial Carcinoma (UTUC): A Population-Based Registry Study from Hamburg, Germany (2004–2021)

**DOI:** 10.3390/cancers17172724

**Published:** 2025-08-22

**Authors:** Annemarie Schultz, Niklas Jobst, Frederik Peters, Christopher Netsch, Clemens M. Rosenbaum, Simon Filmar

**Affiliations:** 1 Hamburg Cancer Registry, 20097 Hamburg, Germany; annemarie.schultz@bwfgb.hamburg.de (A.S.); niklas.jobst@bwfgb.hamburg.de (N.J.);; 2 Department of Urology, Asklepios Hospital Barmbek, 22307 Hamburg, Germany; c.netsch@asklepios.com (C.N.); s.filmar@asklepios.com (S.F.); 3 Department of Urology, University Hospital Schleswig-Holstein, 23562 Lübeck, Germany; 4 Department of Urology, Asklepios Paulinen Hospital, 65197 Wiesbaden, Germany

**Keywords:** UTUC, epidemiology, gender, outcome, cancer registry

## Abstract

Background: Urothelial carcinoma is the second most common urological cancer, mainly affecting the bladder (90–95% of cases) while primary Upper Urinary Tract Urothelial Carcinomas (UTUC) are rare (5–10%). Socioeconomic and gender differences are known in urological cancers like urothelial carcinoma of the bladder, often based on national indices rating cities as single units. This study investigated factors influencing survival in patients with primary UTUC within Hamburg, Germany. Methods: We conducted a retrospective population-based cohort study using data extracted from the Hamburg cancer registry for all primary UTUC cases diagnosed between January 2004 and June 2021. Patient and tumor characteristics, socioeconomic status (measured by a neighborhood-level deprivation index), treatment patterns, and survival outcomes were analyzed. Kaplan–Meier analyses estimated survival probabilities, and a Cox Proportional Hazard Model with and without time transformation assessed survival factors. Results: The cohort included 727 patients (median age 74, 42.2% female), with a median follow-up of 2.2 years (IQR: 0.8–5.5 years). Relevant survival predictors were age, sex, and cancer stage. Older age was associated with reduced excess mortality risk (HR = 0.974), while female sex (HR = 1.472) and advanced stage (HR = 3.343) were associated with higher excess mortality risk. Socioeconomic status and diagnosis period had no measurable impact. Conclusions: Yet, a small sample size and single registry data may limit the generalizability of these results. Further research with larger cohorts is needed.

## Simple Summary

People with fewer social and economic resources often have lower chances of early cancer detection and access to effective treatment. This study investigates how living conditions in different neighborhoods of Hamburg—measured using a deprivation index—affect the survival chances of patients with a cancer of the upper urinary tract. Using data from over 700 patients recorded in the Hamburg Cancer Registry, the study aims to understand whether social inequality influences cancer outcomes. The goal is to help identify ways to improve care and support for all patients, regardless of their socioeconomic background, and to reduce health disparities in cancer treatment and survival.

## 1. Introduction

The association between socioeconomic status (SES) and cancer outcomes has been a topic of extensive research due to its potential to uncover health disparities across different demographic groups [1,2,3]. SES, which reflects the social and economic conditions in which individuals live, plays a critical role in determining access to healthcare, treatment opportunities, and ultimately cancer survival [4]. In Germany, the integration of the new federal cancer registration law, effective since 2013, has made it possible to systematically track cancer diagnoses, treatments, progressions, and outcomes on a national scale [5]. This legislation allows for a detailed, population-based approach to cancer surveillance, enabling research into the impact of various socioeconomic factors on health outcomes.

UTUC, a rare but aggressive form of cancer that affects the renal pelvis and ureter, has a particularly poor prognosis in advanced stages. The guidelines by the European Association of Urology (EAU) divide UTUC into low- and high-risk disease [6]. Regarding the risk stratification, overall and cancer-specific survival differs a lot. Van Doeveren et al. investigated an extensive Dutch cohort of upper tract urothelial carcinoma and reported five-year cancer-specific survival (CSS) rates of 86% for tumors confined to the mucosa or submucosa (non-muscle-invasive), 70% for muscle-invasive tumors without extraurothelial extension, and 44% for cases exhibiting local advancement beyond the organ confines [7].

In contrast, recent population-based data from the SEER registry focusing on patients undergoing radical nephroureterectomy (RNU) for high-risk UTUC revealed stage-specific five-year CSS estimates: 86% for T1N0 disease, 77% for T2N0, 63% for T3N0, and a markedly reduced survival of 39% for either T4N0 or any tumor stage with regional lymph node metastases (N1–2) [8]. Previous research has indicated that individuals from lower SES backgrounds often face barriers to early diagnosis, to access to treatment, and to appropriate healthcare services, which may influence both survival and treatment outcomes [4,9,10,11]. However, studies on the relationship between SES and UTUC outcomes remain scarce, and those that exist often focus on limited geographic regions or specific treatment modalities [9,12].

In this context, the current study seeks to examine the impact of SES, as determined by the deprivation index of Hamburg’s urban districts, on the survival of UTUC patients. By utilizing a comprehensive dataset from the Hamburg Cancer Registry, which includes data from over 700 primary UTUC cases diagnosed between 2004 and 2021, this research explores the role of SES in the prognosis of UTUC patients, controlling for other key variables such as age, gender, and cancer stage. Understanding the interaction between SES and cancer survival in this context is crucial for informing public health strategies aimed at reducing health disparities and improving outcomes for vulnerable populations.

The primary objective was to assess the impact of area-based socioeconomic deprivation on overall survival and relative survival in UTUC patients. We hypothesized that (1) patients from lower SES neighborhoods would have worse survival outcomes, (2) female patients would present with more advanced disease stages, and (3) access to specialized oncological care would vary by socioeconomic status. Secondary objectives included identifying other prognostic factors and analyzing treatment patterns across different demographic groups.

## 2. Material and Methods

### 2.1. Study Design

We conducted a retrospective population-based cohort study using mandatory cancer registry data from Hamburg, Germany.

### 2.2. Data Source

Enabled by the new federal cancer registration law, the registration of all diagnoses, treatments, progressions, and deaths is mandatory in Germany since 2013 and compensated by public and private health insurance funds [13]. Among all federal states, Hamburg was among the first to integrate this new system in its already running epidemiological registration of cancer data.

The Hamburg Cancer Registry, established in the late 1920s and continuously operating since 1985, maintains estimated completeness >90% during 2013–2018, meeting national criteria for inclusion in Germany’s reference region for quality assessment [5].

The city of Hamburg is divided into 103 urban districts, and for each of them, a deprivation score is available based on unemployment rate, social housing, welfare recipients, house/apartment size, and household income, which will be used as a proxy for area-based socio-economic status (SES) below grouped into low, middle, and high according to terciles of cases in the general population [14,15,16]. The registry covers all cancer cases in the population of Hamburg (1.85 million inhabitants in 2020) with mandatory reporting by state law for hospitals, physician offices, and pathology institutions. Data quality follows the Bray and Parkin framework with histological verification >95% and death certificate-only cases <15% [17]. Data on background mortality, necessary for computing relative survival, were extracted from German life tables provided by the Federal Statistical Office.

Germany has a universal, multi-payer healthcare system that provides comprehensive medical coverage to all residents. The majority of the population is covered by Statutory Health Insurance (SHI), while a smaller portion holds Private Health Insurance (PHI). Patients do not typically pay directly for their care; instead, they pay monthly insurance contributions. Most medical services are covered. Privately insured patients usually pay upfront and are reimbursed later. The system is organized into outpatient care, provided by office-based general practitioners and specialists, and inpatient care, delivered by a mix of public, private nonprofit, and private for-profit hospitals. All hospitals that are part of the statutory system are regulated and reimbursed similarly. Access to care, including at university hospitals or private hospitals, is generally not difficult. University hospitals are accessible to all patients. Private hospitals may prioritize patients with private insurance, but in principle, access is based on medical need. Access to urology departments or cancer care is typically straightforward, especially for urgent or oncological cases, which are prioritized. With a referral from a general practitioner or specialist, patients usually do not face major barriers to receiving specialized care. Oncological centers are certified centers that meet strict quality standards defined by the German Cancer Society. They ensure interdisciplinary collaboration, guideline-based treatment, and continuous quality improvement for optimal cancer care. However, certification is not mandatory to treat cancers patients in Germany.

### 2.3. Patients

**Figure 1:**
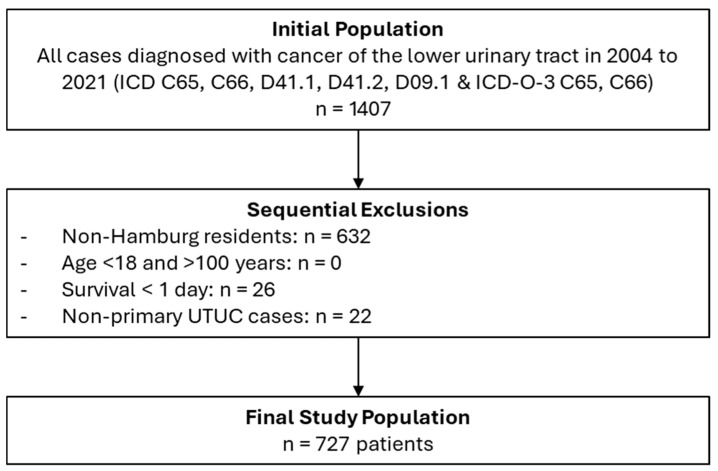
Exclusion and Inclusion Criteria Used to Develop the Final Analytic UTUC-Cohort.

Population-based data on primary UTUC cases (International Classification of Diseases (ICD) C65, C66, D41.1, D41.2, D09.1 (+ICD-O-3 C65, C66)) diagnosed between 2004 and 2021 were extracted from the Hamburg Cancer Registry, which covered 1.85 million inhabitants in 2020 (14). The registry covers all cancer cases in the population of Hamburg. Reporting is mandatory by state law for hospitals, physician offices, and pathology institutions. We excluded cases of patients younger than 18 and older than 100 years old, non-Hamburg residents, and cases where the interval between date of diagnosis and death was <1 day (Figure 1). Cases with missing or invalid data for date of diagnosis, age, gender, or vital status are generally excluded by plausibility checks before entry in the registry database.

### 2.4. Study Variables

A deprivation score for Hamburg (“Sozialindex”) was used as a measure of area-based socioeconomic deprivation on the level of the 103 urban districts in Hamburg [15]. The index is based on official statistics on the unemployment rate, social housing, welfare recipients, house/apartment size, and household income. Two versions were available based on statistics from 2011 to 2020, respectively. We assigned the index to the patients according to the urban district of residence at the time of diagnosis, using the index that is closest to the year of diagnosis (2011 for 2004–2014, 2020 for 2015–2021) [18]. Patients were categorized into three SES groups based on population-based quartiles of the deprivation index: low (1st quartile, 25%), intermediate (2nd and 3rd quartiles combined, 50%), and high (4th quartile, 25%). Based on the categorization of the quartiles, three groups (low, intermediate, and high SES) were defined for our analyses (Table 1).

The time period 2020–2021 was included to analyze potential disruptions in the healthcare system due to the COVID-19 pandemic.

Cancer stage was classified following the Union for International Cancer Control (UICC) TNM classification of malignant tumors effective at the time of diagnosis. Patients without information on stage were excluded from respective analyses.

### 2.5. Missing Data

Missing data patterns were analyzed descriptively. The UICC stage had missing values in 18.2% of the cases and was handled using case-wise deletion in stage-specific analyses.

### 2.6. Outcome

The primary outcome of the study was overall survival. Ten-year survival rates were extracted for descriptive comparison, though follow-up extended beyond 10 years for patients diagnosed in earlier years. Patients diagnosed between 2004 and 2021 were followed until the date of death, emigration from Hamburg, or 31 December 2022, whichever occurred first. Follow-up was based on mandatory linkage with population registries and death certificate data, ensuring 100% completeness of vital status information through December 2022.

### 2.7. Statistical Analysis

Descriptive statistics were presented using proportions for discrete variables and mean or median for continuous variables. Baseline characteristics were described using summary statistics stratified by SES and gender. To avoid multiple testing issues and given the descriptive nature of the baseline comparisons, formal statistical testing was not performed. Potential confounding by baseline differences was addressed through multivariable Cox regression modeling. Unadjusted overall survival by SES group was computed by Kaplan–Meier functions with the log-rank test.

For calculating adjusted overall survival data, two complementary approaches were employed. First, a Cox Proportional Hazards Model with log-transformation of survival time was fitted using the rstrans function from the relsurv package in R to estimate relative survival [19]. This approach transforms survival time to account for expected background mortality derived from German population life tables by age, sex, and calendar year, providing estimates that approximate cancer-specific survival. Second, a standard Cox Proportional Hazards Model was fitted to model the overall survival of the UTUC cohort. We report estimated adjusted hazard ratios with 95% confidence intervals. Low SES served as the reference category consistent with our a priori hypothesis that patients from lower SES neighborhoods would have worse survival outcomes, allowing the direct interpretation of hazard ratios as protective effects of higher socioeconomic status. Visual model diagnostics based on standardized Schoenfeld residuals were performed to ensure the validity of the proportional hazard’s assumption. A two-sided significance level of α = 0.05 was used, with no adjustment for multiple testing due to the exploratory nature of subgroup analyses.

### 2.8. Sample Size and Power

This was an observational study using all available registry cases; no formal power calculation was performed a priori. Analyses were conducted in R, version 4.3.1 (Vienna, Austria, 2024). Furthermore, we followed the Strengthening the Reporting of Observational Studies in Epidemiology (STROBE) statement (Supplementary File S1) [20].

## 3. Results

Of 1407 initially identified UTUC cases, 727 patients (42.4% females, median age 74, median follow-up 2.2 years, Table 1) met the inclusion criteria and comprised the final analytical cohort (Figure 1).

Among the male patients (*n* = 420), the SES distribution was as follows: 74 patients (17.6%) low SES, 223 patients (53.1%) intermediate SES, and 123 patients (29.3%) high SES. Among the female patients (*n* = 307), the distribution was the following: 58 patients (18.9%) low SES, 180 patients (58.6%) intermediate SES, and 69 patients (22.5%) high SES. The median age was similar across SES groups (72–77 years), with females being slightly older than males across all SES categories. The median age at diagnosis varies slightly across groups, with values ranging from 72 to 77 years. The age distribution indicates most patients being in the older age groups (65–74 and 75+).

The distribution of cancer stages (0, I or II, III or IV) shows a higher percentage of advanced stages (III or IV) in all groups. In general, female patients with low and intermediate SES were more often diagnosed with higher UICC stages III or IV than female patients with high SES (62.1% and 55.0% versus 43.5%). Notably, among the male patients, no trend toward a more favorable stage distribution in less deprived regions was observed. There is a notable percentage of cases where the UICC stage is not available, particularly for female patients with high SES (26.1%).

The study also analyzed the rates of systemic therapy among different socioeconomic and gender groups (Table 1).

Systemic therapy rates varied by gender and SES: males showed minimal SES-related variation (14.6–16.1%), while females demonstrated a clear SES gradient (6.9% low vs. 15.9% high SES). These findings indicate variations in the administration of systemic therapy based on socioeconomic status and gender.

As illustrated in Table 1, the study analyzed the rates of resecting surgery and treatment in oncological centers among different socioeconomic and gender groups. For resecting surgery, males showed modest variation across SES groups (16.2% low, 22.9% intermediate, and 26.8% high SES). Among the females, surgical access appeared similar across socioeconomic strata (25.9% low, 23.9% intermediate, and 23.2% high SES), with minimal clinically meaningful differences. Regarding treatment in oncological centers, 6.8% of males with low SES, 11.7% with intermediate SES, and 14.6% with high SES received treatment in these specialized centers. For females, the percentages were 10.3% for low SES, 7.2% for intermediate SES, and 15.9% for high SES. These findings highlight disparities in access to resecting surgery and specialized oncological care based on socioeconomic status and gender. It is important to note that these percentages are based on relatively small case numbers, which may affect the robustness of the results.

The mortality among males was 70.3% for low SES, 66.8% for intermediate SES, and 65.0% for high SES (Table 1). For females, mortality was 70.7% for low SES, 73.9% for intermediate SES, and 65.2% for high SES. The median follow-up period, expressed in days, varies across groups, with values ranging from 625.5 to 966 days.

UICC stage information was missing in 132 patients (18.2%), with no systematic pattern by SES group (*p* = 0.47).

**Figure 2:**
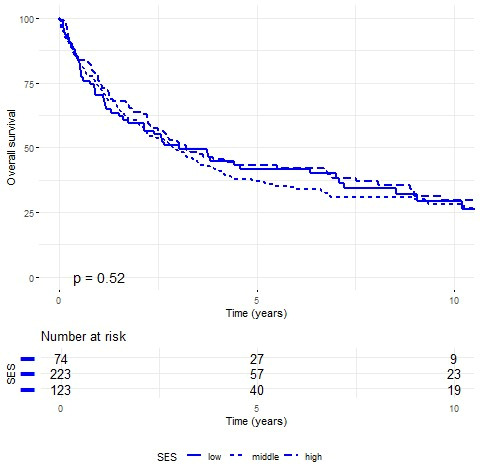
Kaplan–Meier survival curves for male patients by socioeconomic status (SES). Ten-year survival rates: low SES 29.40%, intermediate SES 27.97%, high SES 29.51% (log-rank *p* = 0.52).

**Figure 3:**
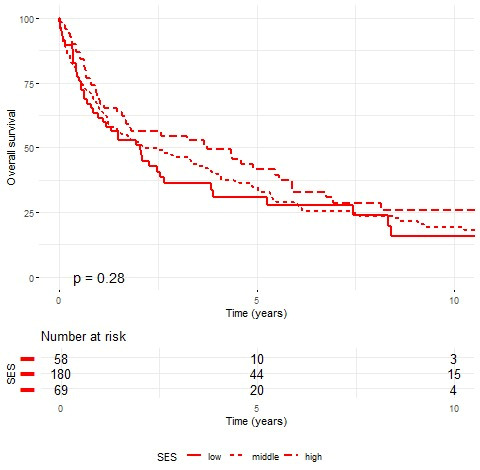
Kaplan–Meier survival curves for female patients by socioeconomic status (SES). Ten-year survival rates: low SES 15.81%, intermediate SES 19.20%, and high SES 25.89% (log-rank *p* = 0.28).

Kaplan–Meier analysis revealed no statistically significant survival differences by SES in either gender (Figure 2 and Figure 3). Males showed similar survival across SES groups (~29%), while females demonstrated a socioeconomic gradient (25.9% high vs. 15.8% low SES). Yet, the *p*-values of 0.52 for male cases and 0.28 for female cases indicate no statistically significant differences in survival rates among the SES groups for either gender. One-year survival rates were 73.7% for males (70.3% low, 73.8% intermediate, and 75.6% high SES) and 65.6% for females (61.5% low, 65.4% intermediate, and 69.6% high SES). Five-year survival rates were 39.9% for males (41.7% low, 37.1% intermediate, and 43.4% high SES) and 35.5% for females (30.8% low, 34.5% intermediate, and 41.6% high SES).

Table 2 presents the results of both standard Cox proportional hazards models and transformed time models expressing relative survival.

Overall Survival: The standard Cox model revealed that older age was associated with increased mortality risk (HR = 1.034 per year, 95% CI: 1.023–1.046, *p* < 0.001). Female sex showed no significant association with survival (HR = 1.053, 95% CI: 0.863–1.284, *p* = 0.612). Advanced UICC stage III/IV was strongly associated with poor prognosis (HR = 3.326, 95% CI: 2.105–5.253, *p* < 0.001). Neither intermediate SES (HR = 0.876, 95% CI: 0.678–1.132, *p* = 0.311) nor high SES (HR = 0.802, 95% CI: 0.595–1.080, *p* = 0.146) showed significant associations with survival. The diagnosis period 2020–2021 had no significant impact (HR = 1.092, 95% CI: 0.781–1.526, *p* = 0.607).

Relative Survival: The transformed time model, which adjusts for background mortality and competing risks, revealed different patterns for age and sex associations. Older age was associated with reduced excess mortality risk (HR = 0.974 per year, 95% CI: 0.963–0.985, *p* < 0.001). Female sex emerged as a significant predictor of excess mortality (HR = 1.463, 95% CI: 1.197–1.788, *p* < 0.001). An advanced UICC stage remained a strong predictor (HR = 3.282, 95% CI: 2.077–5.184, *p* < 0.001). SES associations remained non-significant in both intermediate (HR = 0.876, 95% CI: 0.677–1.133, *p* = 0.313) and high SES groups (HR = 0.802, 95% CI: 0.595–1.080, *p* = 0.146). Interaction testing between SES and gender revealed no significant interaction effect (*p* = 0.81), indicating that the association between SES and survival does not differ significantly between men and women, despite the observed descriptive differences in survival patterns across socioeconomic strata.

## 4. Discussion

This population-based study did not uncover socioeconomic differences in UTUC. Yet, age was associated with worse overall survival while female sex was associated with worse relative survival.

Diagnosis and risk stratification are cornerstones of therapeutic decisions in the management of patients with upper tract urothelial carcinoma (UTUC) [21,22]. The data presented in this study provide insights into the characteristics of UTUC cases in Hamburg, differentiated by socio-economic status (SES) and gender. Thus, this may help to improve diagnostics across all patient groups [23].

### 4.1. Socio-Economic Status and Gender

The observed SES distribution aligns with previous findings in urothelial cancers [24]. Female patients from lower SES backgrounds were more frequently diagnosed with advanced-stage disease, consistent with delayed presentation patterns observed in other malignancies [1,2,10].

For male patients, no clear trend towards a more favorable stage distribution in less deprived regions was observed. These data are complementary to findings on urothelial cancer of the bladder in Hamburg [14]. This demonstrates that gender and SES have, for example, a high impact on female patients, on diagnosis and treatment in several urological malignancies.

Not surprisingly, the data shown in this paper again emphasize the impact on female patients with a higher risk stratification compared to their male counterparts [21,22]. This aligns with findings from other malignancies [1,2,4,25].

Regardless of the SES, a higher percentage of advanced stages (III or IV) was observed across all groups. The distribution of UICC stages reveals that 48.7% of patients were diagnosed at advanced stages (III or IV). This emphasizes that UTUC represents an aggressive rare malignancy, which is difficult to detect in an early stage. Clinical symptoms like gross hematuria, pain or Hydronephrosis are often presenting in an advanced stage rather than in lower stages [26,27]. This differentiates UTUC from urothelial cancer of the bladder which has a higher likelihood of getting detected by, e.g., microhematuria in urine testing [10,28].

### 4.2. Access to Systemic Therapy and Specialized Care

The study further reveals disparities in access to systemic therapy and surgery, stratified by SES and gender. Female patients from lower SES backgrounds demonstrated reduced access to both systemic therapy and specialized oncological care, while male patients showed minimal SES-related treatment variations.

These findings are consistent with Seisen et al., who reported socioeconomic and insurance-based disparities in the application of neoadjuvant chemotherapy among UTUC patients in the U.S. [6]. Although Germany provides more universal healthcare access, these data suggest that non-financial barriers—such as geographic distance, health literacy, and referral patterns—still play a role.

In contrast, male patients showed minimal variation in systemic therapy rates across SES groups, though access to specialized centers remained SES-dependent. Surgical access appeared generally equitable across socioeconomic groups, particularly among women, with minimal clinically meaningful differences in resecting surgery rates.

### 4.3. Treatment

Surgical rates showed modest variation across groups, with access to resecting surgery appearing generally equitable across socioeconomic strata, particularly among women.

Overall survival remained poor, reflecting UTUC’s aggressive nature and consistent with survival rates reported in other population-based series [7,8]. Women demonstrated a socioeconomic survival gradient, while men showed minimal SES-related differences, a pattern that mirrors gender-specific healthcare disparities observed in other malignancies [1,2,4].

Interestingly, the diagnosis period (2020–2021) did not significantly impact survival (HR: 1.154; *p* = 0.395). In comparison to bladder cancer, Garg et al. could show that there is a significant delay for the female gender in diagnosis than in their male counterparts [10], which is one reason why female patients are thought to present with advanced stages. This could be interpreted as indicating that UTUC is an aggressive cancer presenting often with metastasis so that the diagnosis period does not play any role in the overall survival. Moreover, the time period may indicate that, despite potential disruptions in the healthcare system due to the COVID-19 pandemic, cancer treatment in Hamburg remained largely stable.

### 4.4. Survival Outcomes

Despite disparities in tumor stage and access to care, Kaplan–Meier analysis revealed no statistically significant survival differences by SES in either gender. However, the pattern of associations differed markedly between men and women. Males showed virtually identical survival across all SES groups, suggesting that socioeconomic factors may have limited influence on male UTUC outcomes. In contrast, females demonstrated the expected socioeconomic gradient with a 10% absolute difference between high and low SES groups, though this did not reach statistical significance (*p* = 0.28). This gender-specific pattern may reflect differential healthcare-seeking behaviors, treatment compliance, or access patterns between men and women, even within a universal healthcare system. These results partially contrast with a well-established association between low SES and poorer cancer outcomes [12]. Several explanations may account for the findings: (1) UTUC’s aggressive nature may mask socioeconomic associations, (2) Germany’s universal healthcare system may attenuate SES-related associations with outcomes, (3) the area-based SES measure may not capture individual-level socioeconomic factors, (4) the study may be underpowered to detect modest SES effects, and (5) unmeasured confounders (comorbidities, performance status) may mediate the SES-survival relationship.

Notably, the survival differences between men and women varied by SES group, suggesting a potential interaction between gender and socioeconomic status. Among low and intermediate SES groups, women showed markedly worse survival compared to men, while this gender gap was less pronounced in the high SES group. This pattern may reflect differential access to specialized care, healthcare-seeking behaviors, or treatment compliance across socioeconomic strata, even within Germany’s universal healthcare system.

The comparison between Overall and Relative Survival provides important insights into the interpretation of survival associations in elderly cancer populations. The standard Cox model revealed expected patterns with older age associated with increased mortality risk (HR = 1.034, *p* < 0.001) and no significant sex differences (HR = 1.053, *p* = 0.612). However, the transformed time model, which adjusts for background mortality, showed different patterns: older age was associated with reduced excess mortality risk (HR = 0.974, *p* < 0.001), while female sex emerged as a significant predictor of poor prognosis (HR = 1.463, *p* < 0.001). Advanced UICC stage III/IV remained consistently associated with poor outcomes in both models (HR = 3.326 and 3.282, respectively, both *p* < 0.001). These findings demonstrate that both overall and relative survival need to be taken into account when interpreting prognostic factors, particularly age and sex, in elderly cancer populations where competing risks play a substantial role.

### 4.5. Limitations

Our study has several limitations. First, the relatively small sample size, particularly in SES subgroups (*n* = 58–223), may limit statistical power to detect clinically meaningful differences and reduces generalizability. Second, the area-based SES measure may not reflect individual socioeconomic circumstances. Fourth, competing risks from comorbidities were not formally modeled, potentially biasing survival estimates in this elderly population. Finally, unmeasured confounders including comorbidity burden, social support, and healthcare-seeking behavior may influence both SES and survival outcomes. Our observational design precludes causal inferences about the relationships between patient characteristics and survival outcomes.

These findings from Hamburg, a metropolitan area with universal healthcare coverage, may not generalize to healthcare systems with greater access barriers or rural populations with different referral patterns. The relatively high proportion of advanced-stage disease (48.7% stage III/IV) is consistent with other UTUC series, supporting the external validity of our cohort. However, treatment patterns and survival outcomes may differ in other German regions or international settings. Nevertheless, our methodological approach has important strengths. The transformed time approach is particularly valuable in this elderly population where competing risks from non-cancer mortality may confound overall survival analyses. This method specifically addresses competing risks from cardiovascular disease, other cancers, and age-related conditions. As comorbidity data are not systematically collected in cancer registries, our use of population-based life tables provides the best available adjustment for background mortality risk.

## 5. Conclusions

Our findings underscore the importance of age, sex, and cancer stage at diagnosis as critical factors influencing survival outcomes. While we found no significant impact of area-based SES on survival, this may reflect UTUC’s aggressive nature, universal healthcare access in Germany, or methodological limitations rather than definitively excluding socioeconomic influences. The consistently poor survival across all SES groups emphasizes the need for improved early detection strategies and novel therapeutic approaches for this aggressive malignancy. Early detection and tailored treatment strategies, particularly for high-risk groups identified by sex and cancer stage, are essential for improving patient outcomes.

## Figures and Tables

**Table 1:** Characteristics of UTUC Cases by Socioeconomic Deprivation Group and Gender in Hamburg.

		Male			Female		
	Overall	Low	Intermediate	High	Low	Intermediate	High
*n*	727	74	223	123	58	180	69
share of *n*		17.6%	53.1%	29.3%	18.9%	58.6%	22.5%
period of diagnosis = 2004–2019, *n* (%)	628 (86.4)	69 (93.2)	193 (86.5)	110 (89.4)	48 (82.8)	147 (81.7)	61 (88.4)
= 2020–2021, *n* (%)	99 (13.6)	5 (6.8)	30 (13.5)	13 (10.6)	10 (17.2)	33 (18.3)	8 (11.6)
sex = female (%)	307 (42.2)	0 (0.0)	0 (0.0)	0 (0.0)	58 (100.0)	180 (100.0)	69 (100.0)
age group (%)							
18–44	5 (0.7)	2 (2.7)	1 (0.4)	1 (0.8)	0 (0.0)	0 (0.0)	1 (1.4)
45–54	37 (5.1)	5 (6.8)	9 (4.0)	6 (4.9)	5 (8.6)	10 (5.6)	2 (2.9)
55–64	92 (12.7)	12 (16.2)	36 (16.1)	16 (13.0)	9 (15.5)	15 (8.3)	4 (5.8)
65–74	247 (34.0)	25 (33.8)	73 (32.7)	47 (38.2)	13 (22.4)	62 (34.4)	27 (39.1)
75+	346 (47.6)	30 (40.5)	104 (46.6)	53 (43.1)	31 (53.4)	93 (51.7)	35 (50.7)
age at diagnosis (median [IQR])	74.00 [67.00–80.00]	72.00 [64.25–77.75]	74.00 [66.00–79.50]	73.00 [67.00–79.50]	77.00 [66.25–81.75]	75.00 [68.00–81.00]	75.00 [69.00–79.00]
UICC stage (%)							
0	46 (6.3)	8 (10.8)	14 (6.3)	8 (6.5)	4 (6.9)	5 (2.8)	7 (10.1)
I or II	195 (26.8)	19 (25.7)	72 (32.3)	32 (26.0)	10 (17.2)	48 (26.7)	14 (20.3)
III or IV	354 (48.7)	32 (43.2)	98 (43.9)	59 (48.0)	36 (62.1)	99 (55.0)	30 (43.5)
NA	132 (18.2)	15 (20.3)	39 (17.5)	24 (19.5)	8 (13.8)	28 (15.6)	18 (26.1)
systemic therapy = yes (%)	101 (13.9)	11 (14.9)	36 (16.1)	18 (14.6)	4 (6.9)	21 (11.7)	11 (15.9)
resecting = yes (%)	170 (23.4)	12 (16.2)	51 (22.9)	33 (26.8)	15 (25.9)	43 (23.9)	16 (23.2)
oncological center = yes (%)	79 (10.9)	5 (6.8)	26 (11.7)	18 (14.6)	6 (10.3)	13 (7.2)	11 (15.9)
vital status = dead (%)	500 (68.8)	52 (70.3)	149 (66.8)	80 (65.0)	41 (70.7)	133 (73.9)	45 (65.2)
follow up (median [IQR])	809.00 [288.50–2000.00]	946.50 [293.75–2624.25]	812.00 [327.50–1882.50]	966.00 [369.00–2461.50]	625.50 [189.25–1234.25]	660.00 [189.25–1767.25]	929.00 [300.00–2030.00]

**Table 2:** Overall Survival and Relative Survival for UTUC Cases in Hamburg.

	Overall Survival				Relative Survival			
Variable	HR	95% CI Lower	95% CI Upper	*p*-Value	HR	95% CI Lower	95% CI Upper	*p*-Value
Period of diagnosis: 2020–2021	1.092	0.781	1.526	0.607	1.142	0.817	1.595	0.437
Age (per year)	1.034	1.023	1.046	<0.001	0.974	0.963	0.985	<0.001
Female sex	1.053	0.863	1.284	0.612	1.463	1.197	1.788	<0.001
UICC stage I or II	1.080	0.665	1.756	0.755	1.114	0.686	1.807	0.663
UICC stage III or IV	3.326	2.105	5.253	<0.001	3.282	2.077	5.184	<0.001
SES intermediate	0.876	0.678	1.132	0.311	0.876	0.677	1.133	0.313
SES high	0.802	0.595	1.080	0.146	0.802	0.595	1.080	0.146

HR = Hazard Ratio; CI = Confidence Interval. Reference categories: Period 2004–2019, Male sex, UICC stage 0, SES low. Standard Cox model assumes proportional hazards; Transformed time model accounts for competing risks using German population life tables.

## Data Availability

The data presented in this study are available on request from the corresponding author.

## Supplementary Materials

The following supporting information can be downloaded at: https://www.mdpi.com/article/10.3390/cancers17172724/s1, File S1: STROBE Checklist for Cohort Studies.
