# Supplementary material for: The Impact of Patient, Tumor, and Socioeconomic Characteristics on Survival in Upper Urinary Tract Urothelial Carcinoma (UTUC): A Population-Based Registry Study from Hamburg, Germany (2004–2021)
Source: Cancers (Basel). 2025 Aug 22;17(17):2724. doi: 10.3390/cancers17172724 (PMC12427197; doi:10.3390/cancers17172724)
Supplement: Supplementary file 1 [file cancers-17-02724-s001.zip › cancers-3721856-supplementary.pdf]

**STROBE Checklist for Cohort Studies**

**Title:** The Impact of Patient, Tumor, and Socioeconomic Characteristics on Survival in Upper Urinary Tract Urothelial Carcinoma (UTUC): A Population-Based Registry Study from Hamburg, Germany (2004-2021)

**Title and Abstract**

| Item | Recommendation | Page | Location in Manuscript |
|---|---|---|---|
| 1 | (a) Indicate the study's design with a commonly used term in the title or the abstract | 1 | Title: "Population-Based Registry Study" |
| | (b) Provide in the abstract an informative and balanced summary of what was done and what was found | 1 | Abstract: Comprehensive summary including background, methods, results, and conclusions |

**Introduction**

| Item | Recommendation | Page | Location in Manuscript |
|---|---|---|---|
| 2 | Explain the scientific background and rationale for the investigation being reported | 2-3 | Introduction: SES and cancer outcomes background, UTUC characteristics, research gap |
| 3 | State specific objectives, including any prespecified hypotheses | 3 | End of Introduction: Primary and secondary objectives with three specific hypotheses |

**Methods**

| Item | Recommendation | Page | Location in Manuscript |
|---|---|---|---|
| 4 | Present key elements of study design early in the paper | 4 | Methods: "Retrospective population-based cohort study" |
| 5 | Describe the setting, locations, and relevant dates, including periods of recruitment, exposure, follow-up, and data collection | 4-5 | Data source section: Hamburg Cancer Registry 2004-2021, follow-up until December 2022 |
| 6 | (a) Give the eligibility criteria, and the sources and methods of selection of participants | 5 | Patients section: ICD codes, inclusion/exclusion criteria, Figure 1 |

| Item | Recommendation | Page | Location in Manuscript |
|---|---|---|---|
| | (b) For matched studies, give matching criteria and number of exposed and unexposed | N/A | Not applicable - not a matched study |
| 7 | Clearly define all outcomes, exposures, predictors, potential confounders, and effect modifiers. Give diagnostic criteria, if applicable | 5-7 | Study Variables: SES index definition, cancer staging (UICC), outcome definition (overall survival) |
| 8 | For each variable of interest, give sources of data and details of methods of assessment (measurement). Describe comparability of assessment methods if there is more than one group | 5-7 | Study Variables and Statistical analysis: Hamburg deprivation index, cancer registry data, life tables |
| 9 | Describe any efforts to address potential sources of bias | 7 | Missing Data section, Statistical analysis: case-wise deletion, model diagnostics |
| 10 | Explain how the study size was arrived at | 7 | Sample Size and Power: "observational study using all available registry cases" |
| 11 | Explain how quantitative variables were handled in the analyses. If applicable, describe which groupings were chosen and why | 6-7 | Study Variables: SES tertiles (low/intermediate/high), age groups, UICC staging |
| 12 | (a) Describe all statistical methods, including those used to control for confounding | 7 | Statistical analysis: Cox proportional hazards models, Kaplan-Meier, relative survival |
| | (b) Describe any methods used to examine subgroups and interactions | 7 | Statistical analysis: Stratified by SES and gender |
| | (c) Explain how missing data were addressed | 6-7 | Missing Data section: Case-wise deletion for stage, exclusion of functional status |
| | (d) If applicable, explain how loss to follow-up was addressed | 6 | Outcome section: Population registry linkage ensuring 100% completeness |

| Item | Recommendation | Page | Location in Manuscript |
|---|---|---|---|
| | (e) Describe any sensitivity analyses | 7 | Statistical analysis: Two complementary Cox models (standard and transformed time) |

**Results**

| Item | Recommendation | Page | Location in Manuscript |
|---|---|---|---|
| 13 | (a) Report numbers of individuals at each stage of study—eg numbers potentially eligible, examined for eligibility, confirmed eligible, included in the study, completing follow-up, and analysed | 8 | Results: "Of 1,407 initially identified UTUC cases, 727 patients met inclusion criteria" + Figure 1 |
| | (b) Give reasons for non-participation at each stage | 8 | Figure 1: Exclusion criteria with numbers |
| | (c) Consider use of a flow diagram | 8 | Figure 1: Study flow diagram provided |
| 14 | (a) Give characteristics of study participants (eg demographic, clinical, social) and information on exposures and potential confounders | 8-9 | Table 1: Comprehensive baseline characteristics by SES and gender |
| | (b) Indicate number of participants with missing data for each variable of interest | 9 | Text: "UICC stage information was missing in 132 patients (18.2%)" |
| | (c) Summarise follow-up time (eg, average and total amount) | 8 | Results: "median follow-up of 2.2 years" |
| 15 | Report numbers of outcome events or summary measures over time | 9-10 | Results: Survival rates, mortality data, Kaplan-Meier curves |
| 16 | (a) Give unadjusted estimates and, if applicable, confounder-adjusted estimates and their precision (eg, 95% confidence interval). Make clear which confounders were adjusted for and why they were included | 10-11 | Table 2: Both overall and relative survival models with HR and 95% CI |
| | (b) Report category boundaries when continuous variables were categorized | 6 | Study Variables: SES tertiles definition |

| Item | Recommendation | Page | Location in Manuscript |
| --- | --- | --- | --- |
| | (c) If relevant, consider translating estimates of relative risk into absolute risk for a meaningful time period | 9-10 | Results: 10-year survival rates provided, absolute (overall survival) and relative |

**Discussion**

| Item | Recommendation | Page | Location in Manuscript |
| --- | --- | --- | --- |
| 17 | Summarise key results with reference to study objectives | 11-12 | Discussion: "This population-based study did not uncover socioeconomic differences in UTUC..." |
| 18 | Discuss limitations of the study, taking into account sources of potential bias or imprecision. Discuss both direction and magnitude of any potential bias | 15-16 | Limitations section: Sample size, missing data, area-based SES measure, competing risks |
| 19 | Give a cautious overall interpretation of results considering objectives, limitations, multiplicity of analyses, results from similar studies, and other relevant evidence | 16 | Conclusions: Balanced interpretation with caveats about generalizability |
| 20 | Discuss the generalisability (external validity) of the study results | 15-16 | Limitations: "findings from Hamburg...may not generalize to healthcare systems with greater access barriers" |

**Other Information**

| Item | Recommendation | Page | Location in Manuscript |
| --- | --- | --- | --- |
| 21 | Give the source of funding and the role of the funders for the present study and, if applicable, for the original study on which the present article is based | 17 | Funding: "This research received no external funding" |
| 22 | Other | 17 | Ethics statement, informed consent, data availability, conflicts of interest all addressed |
